# Bacterial-based cancer therapy: mechanisms and therapeutic advances

**DOI:** 10.1186/s43556-026-00524-2

**Published:** 2026-07-30

**Authors:** Arman H. Sharifi, Ngoc Hai Trieu Phong, Anjali Marek, Mohammed A. Kamal, Suendus Al-Kodmany, Duy Binh Tran, Tohru Yamada

**Affiliations:** 1 Department of Surgery, Division of Transplant Surgery, University of Illinois College of Medicine, Chicago, IL 60612 USA; 2 Department of Surgery, Division of Surgical Oncology, University of Illinois College of Medicine, Chicago, IL 60612 USA; 3 College of Liberal Arts and Sciences, University of Illinois Chicago, Chicago, IL 60607 USA; 4 Richard & Loan Hill Department of Biomedical Engineering, University of Illinois College of Engineering, Chicago, IL 60607 USA; 5 Universal School, Bridgeview, IL 60455 USA

**Keywords:** Engineered bacteria, Oncolytic bacteria, Tumor microenvironment, Enzyme–prodrug therapy, Bacterial metabolites, Cancer immunotherapy

## Abstract

Targeted cancer therapies increasingly require platforms that can penetrate poorly perfused tumor regions while minimizing systemic toxicity. Bacteria, owing to their intrinsic tumor tropism, genetic programmability, and immunostimulatory properties, have re-emerged as versatile anticancer agents, ranging from attenuated tumor-colonizing strains to highly engineered “living therapeutics.” In this review, we synthesize the mechanistic foundations and therapeutic advances of bacterial-based cancer therapy through four major themes. First, we examine foundational mechanisms, including tumor-selective colonization, direct oncolysis and cytotoxicity, activation of innate and adaptive immunity, and remodeling of the tumor microenvironment. Second, we discuss engineering strategies that enable controllable delivery of therapeutic payloads, such as cytokines, antibodies and nanobodies, enzyme-prodrug systems, toxins, and nucleic-acid therapeutics, while also improving biosafety and biocontainment. Third, we evaluate combination strategies integrating bacteria with chemotherapy, radiotherapy, phototherapy, and immunotherapy, with emphasis on how bacteria complement conventional modalities by targeting hypoxic, necrotic, and immunologically refractory tumor niches. Fourth, we summarize translational progress, including representative early-phase clinical experiences, manufacturing challenges, and major safety constraints. We also highlight emerging microbiome-disease databases and computational resources that may support target selection, biomarker discovery, and therapy-response stratification. Current evidence supports bacteria as a promising precision modality, particularly for immunologically “cold” or hypoxic tumors; however, major challenges remain in the predictability of intratumoral distribution, host clearance, genetic stability, and long-term safety. Addressing these barriers through rigorous engineering, standardized manufacturing, and clinically meaningful endpoints will be essential for the next generation of bacterial therapeutics in oncology.

## Introduction

Cancer remains a leading cause of morbidity and mortality worldwide, and therapeutic resistance, incomplete tumor control, and treatment-related toxicity continue to limit the durability of many current treatments. A major challenge in solid-tumor oncology is the inability to achieve effective drug delivery and immune activation within poorly perfused, hypoxic, necrotic, and immunosuppressive tumor regions, which frequently contribute to recurrence, metastasis, and treatment resistance [1–4]. Although chemotherapy, radiotherapy, and immunotherapy have improved outcomes in many malignancies, their efficacy is often constrained by inadequate intratumoral penetration, acquired resistance, and unfavorable tumor microenvironments [5–8]. These limitations have created a strong need for alternative or complementary strategies capable of functioning within treatment-refractory tumor niches.

Bacteria represent a distinctive therapeutic platform in this context. Selected bacterial strains can preferentially localize to biologically permissive tumor regions, proliferate under specific microenvironmental conditions, and serve as vehicles for the localized delivery of therapeutic payloads [9, 10]. Bacterial components can also activate innate immune-sensing pathways, stimulate antitumor immunity, remodel the tumor microenvironment, and create opportunities for synergy with other treatment modalities [9, 11–13]. The concept of using bacteria to treat cancer has a long history, beginning with nineteenth-century observations of tumor regression following bacterial infection and the subsequent work of Busch, Fehleisen, and Coley [14–19]. Interest in this field was later revitalized by recombinant DNA technology and an improved understanding of tumor biology, enabling the development of attenuated and engineered strains with greater therapeutic selectivity and reduced pathogenicity [20–22]. *Bacillus Calmette-Guérin* (BCG), which remains an established treatment for non-muscle-invasive bladder cancer, provides an important clinical precedent, while numerous other bacterial genera have since been investigated as potential anticancer platforms [23, 24].

Renewed interest in bacteria-based cancer therapy has been further accelerated by advances in microbiome science, next-generation sequencing, tumor ecology, and synthetic biology [25–29]. These developments have improved understanding of host–microbe interactions and the spatial heterogeneity of tumors, including variations in oxygenation, vascular access, stromal density, metabolic stress, and immune-cell distribution [30–33]. At the same time, modern engineering approaches have transformed bacteria from naturally tumor-colonizing organisms into programmable living therapeutics capable of integrating tumor targeting, direct cytotoxicity, localized drug activation, immune modulation, and biocontainment within a single biological chassis [3, 8, 34–37]. Consequently, the central question is no longer simply whether bacteria can exert anticancer effects, but which bacterial mechanisms are best matched to particular tumor constraints, how their functions can be controlled in vivo, and which safety, manufacturing, and clinical benchmarks should define successful translation [11, 12, 38, 39]. These advances expand the role of bacteria from simple tumor-colonizing agents to programmable living therapeutics that can integrate targeting, cytotoxicity, immune remodeling, and localized drug activation within a single chassis. Simultaneously, progress in microbiome profiling and tumor biology has reinforced the idea that bacterial therapy may be especially relevant in selected biological contexts, such as immunologically “cold” tumors, lesions with extensive hypoxia or necrosis, and cancers in which poor intratumoral drug distribution remains a major treatment barrier [11, 20, 38, 40–48].

**Fig. 1:**
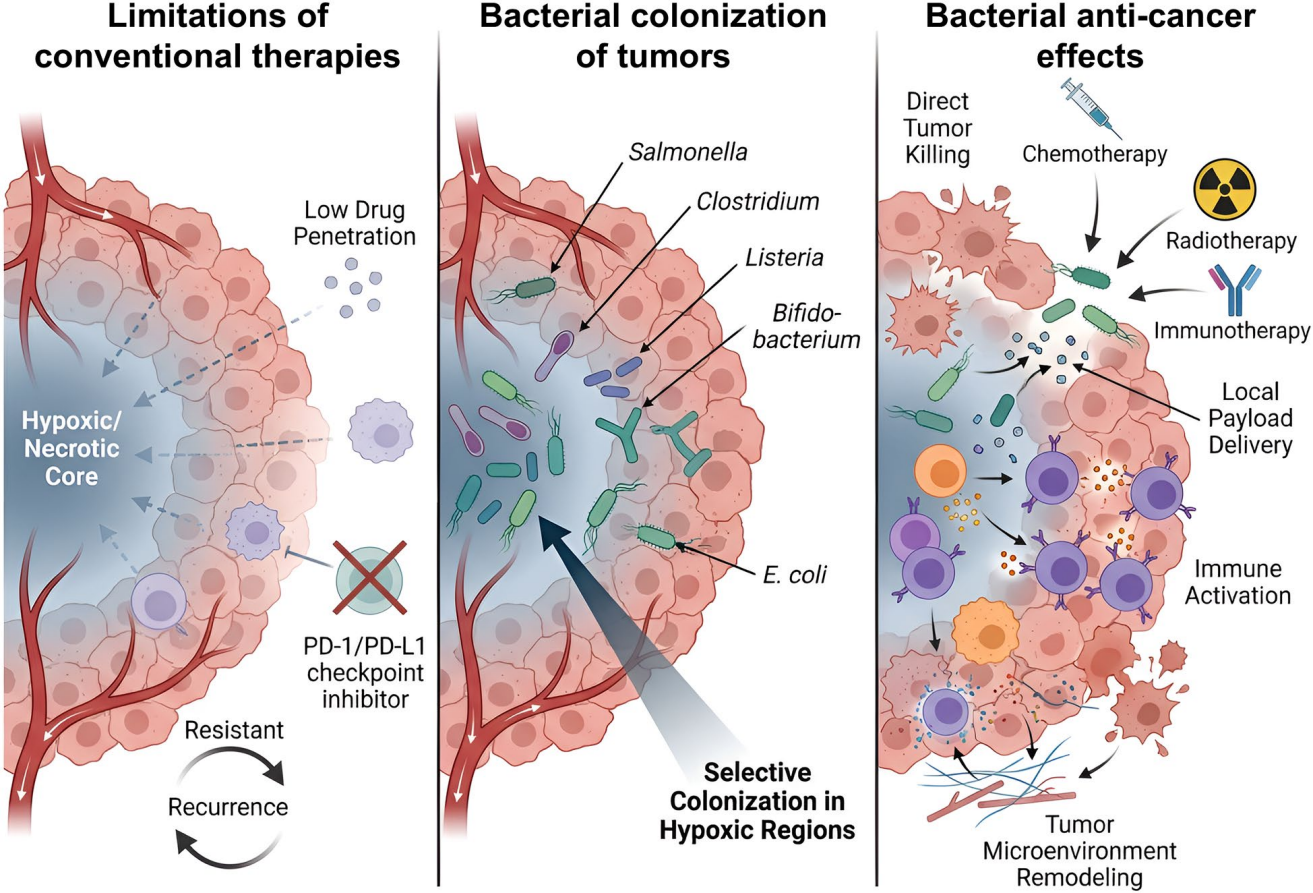
Conceptual overview of bacterial-based cancer therapy. Conventional anti-cancer therapies are often limited by poor drug penetration, hypoxia-associated resistance, and immune suppression within solid tumors. In contrast, tumor-targeting bacteria can preferentially colonize hypoxic and necrotic tumor niches, where they may directly kill cancer cells, deliver therapeutic payloads, activate innate and adaptive immunity, and enhance the efficacy of combination therapies. Created in BioRender, https://BioRender.com

In this review, we examine bacteria-based cancer therapy through four interrelated themes. First, we summarize the foundational mechanisms of bacterial antitumor activity, including tumor-selective colonization, direct cytotoxicity, activation of innate and adaptive immunity, and remodeling of the tumor microenvironment. Second, we review major therapeutic platforms and engineering strategies, including attenuated bacteria, living drug-delivery systems, enzyme–prodrug approaches, immune-active strains, bacterial metabolites and toxins, and synthetic-biology-based designs. Third, we evaluate combination strategies involving chemotherapy, radiotherapy, phototherapy, nanotechnology, and immunotherapy in the context of tumor biology and therapeutic mechanism. Finally, we discuss translational progress, clinical experience, safety and biocontainment, manufacturing considerations, computational resources, and future development priorities. Through this framework, we aim to clarify how bacterial mechanisms can be matched to specific tumor features and translational requirements, thereby defining the design principles needed to advance bacterial therapeutics toward clinically meaningful applications (Fig. 1).

## Foundational mechanisms of bacterial anti-tumor activity

**Fig. 2:**
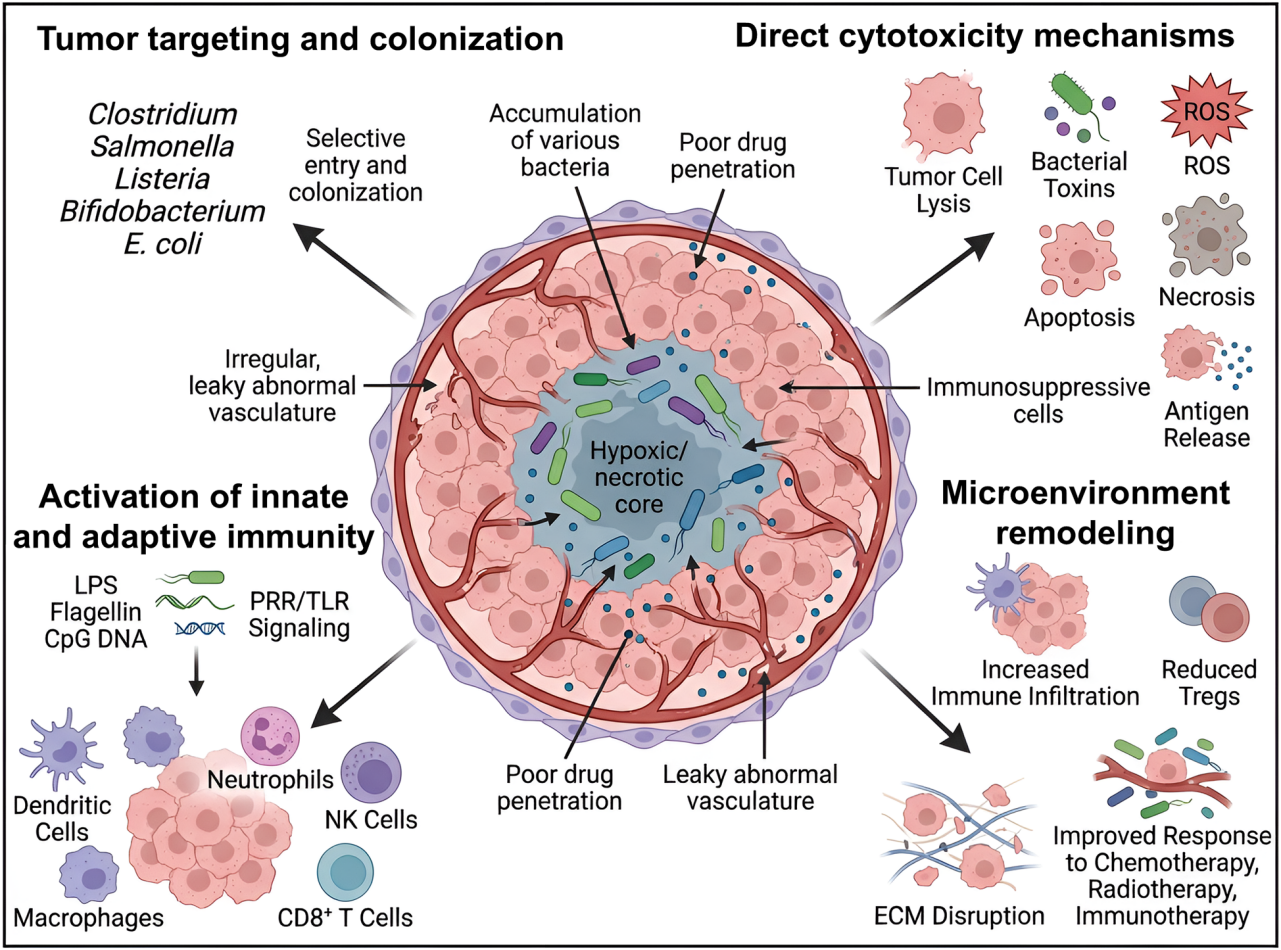
Foundational mechanisms of bacterial anti-tumor activity. Therapeutic bacteria preferentially colonize hypoxic, necrotic, and poorly perfused tumor regions. Once localized, they may directly kill tumor cells through oncolysis, toxin production, reactive oxygen species, and nutrient competition; activate and innate anti-tumor immunity through pathogen-associated molecular pattern recognition and cytokine induction; and remodel the tumor microenvironment by enhancing immune-cell infiltration, reducing immunosuppression, and disrupting ECM barriers. Together, these effects help explain the multifunctional therapeutic potential of bacterial-based cancer therapy. Created in BioRender, https://BioRender.com

Bacterial-based cancer therapy is mechanistically attractive because it exploits biological features of solid tumors that often limit conventional treatment, including hypoxia, necrosis, abnormal vasculature, poor drug penetration, and local immune suppression [34, 49, 50]. Rather than acting through a single pathway, therapeutic bacteria can influence tumor growth through several interconnected mechanisms, including preferential tumor colonization, direct cytotoxicity, activation of innate and adaptive immunity, and remodeling of the tumor microenvironment [10, 34, 49, 51, 52] (Fig. 2). Framing the field around these mechanisms helps distinguish bacterial therapy from passive drug-delivery systems and clarifies why bacterial platforms are often especially effective in combination regimens.

### Tumor targeting and colonization exert direct oncolytic and cytotoxic effects

A major advantage of bacteriotherapy is the ability of certain bacterial species to selectively target the distinctive microenvironment of solid tumors [53, 54]. Many tumors contain poorly perfused, hypoxic, and necrotic regions that are difficult for conventional therapies to reach because of limited vascular supply [53, 55–57]. Obligate anaerobes such as *Clostridium* and *Bifidobacterium* preferentially thrive in these oxygen-deficient niches, where they can colonize and replicate [53, 58]. Facultative anaerobes, including *Salmonella* and *Listeria*, can also accumulate within tumors, driven by factors such as abnormal tumor vasculature, chemotactic responses to tumor-derived compounds, and impaired immune clearance within the immunosuppressive tumor microenvironment [31, 53].

Several factors contribute to this tumor selectivity, including the presence of necrotic tissue, low oxygen tension, abnormal vascular leakiness, nutrient gradients, impaired immune surveillance, and, in some systems, chemotactic responses to tumor-associated metabolites [10, 12, 34, 59]. This preferential colonization is not merely a targeting phenomenon; it forms the basis for downstream therapeutic activity by enabling bacteria to persist within tumors, release therapeutic payloads, and exert direct anti-tumor effects [60, 61].

One illustrative example is the genetically modified strain *S. typhimurium* A1, an auxotrophic mutant that selectively colonizes tumors and directly invades viable cancer cells through its type III secretion system [62]. Once inside the cytoplasm, the bacteria replicate and induce nuclear destruction, leading to tumor cell death and regression [62, 63]. Bacteria exploit tumor-specific markers and adhesion molecules (*e.g.,* integrins like α4β1) to enhance their localization and persistence in tumors [64]. Certain strains may also inhibit tumor growth by competing with cancer cells for essential nutrients [56]. Notably, *S. typhimurium* A1 has been shown to proliferate throughout viable malignant tissue in tumor xenografts, in contrast to earlier bacterial systems that were largely confined to necrotic regions, thereby contributing to tumor inhibition and regression in vivo while remaining absent from normal tissues [65].

As noted above, many bacteria possess intrinsic tumor-targeting properties, particularly anaerobic and facultative anaerobic genera such as *Clostridium*, *Salmonella*, *Listeria*, *E. coli*, and *Bifidobacterium* [66]. These organisms can exploit the hypoxic and immunosuppressed conditions of solid tumors to achieve preferential localization. Besides, our previous studies demonstrated that interactions between cancers, such as breast cancer and melanoma, and bacteria lead to tumor suppression by secreting specific anti-cancer proteins and peptides [67–71]. In some settings, bacteria may also interact with tumor-associated adhesion molecules and other microenvironmental cues that enhance persistence within tumor tissue [64]. For example, attenuated *Salmonella* strains such as VNP20009 can accumulate in tumors at levels reportedly 1,000- to 10,000-fold higher than in normal tissues, while also serving as vehicles for therapeutic gene delivery and immune stimulation [72]. Similarly, *B. longum* can selectively colonize hypoxic tumor regions and has been investigated as a carrier for gene or drug delivery to enhance the efficacy of chemotherapy, radiotherapy, or immunotherapy [73].

In addition to selective localization, some bacterial species exert direct cytotoxic effects. Bacteria can naturally produce toxins, reactive oxygen species, or other oncolytic factors that directly damage tumor cells [74–76]. This cytotoxicity may induce apoptosis or necrosis and can also provoke local inflammation, thereby recruiting immune cells to the tumor site [7, 77]. For example, the anaerobic strain *C. novyi*-NT induces localized tumor necrosis and promotes tumor-antigen release, which may subsequently support adaptive immune activation [56]. Taken together, these findings suggest that tumor targeting and colonization are foundational to bacteria-based cancer therapy, both by enabling selective delivery and by initiating direct anti-tumor injury.

### Activation of innate immunity

Bacteria are potent activators of innate immunity because they carry pathogen-associated molecular patterns (PAMPs), including lipopolysaccharide (LPS), flagellin, peptidoglycan, lipoproteins, and unmethylated cytosine-phosphate-guanine (CpG) DNA. These motifs are recognized by pattern-recognition receptors (PRR), including Toll-like receptors (TLR) and related innate immune sensors expressed by dendritic cells, macrophages, neutrophils, and other immune populations [78–81]. Activation of these pathways can induce inflammatory cytokine production, promote antigen presentation, and recruit effector cells into tumors that were previously poorly inflamed [82–84].

This capacity for innate immune activation is one of the major reasons bacteria are attractive in immunologically “cold” tumors. Unlike many targeted therapies, bacterial systems do not rely solely on tumor cell-intrinsic vulnerabilities; they can also convert the tumor site into a localized inflammatory niche [38, 85]. Recruitment of neutrophils, macrophages, dendritic cells, and natural killer cells may contribute to early tumor control and help establish conditions favorable for adaptive anti-tumor immunity [86].

Upon bacterial colonization, innate immune signaling is initiated through pattern-recognition receptors such as Toll-like receptors, which recognize bacterial components including LPS and flagellin [87]. This activation promotes dendritic-cell maturation, cytokine release, and the recruitment of immune effectors that can directly attack tumor cells or remodel the tumor microenvironment. For example, Toll-like receptor 4 (TLR4) recognizes LPS from Gram-negative bacteria, whereas Toll-like receptor 5 (TLR5) recognizes bacterial flagellin [88]. Consistent with this, *L. monocytogenes* and its derivatives have been shown to enhance T-cell proliferation and cytotoxicity by inducing interleukin-12 (IL-12) and interferon-gamma (IFN-γ) production [89, 90]. However, innate activation is a double-edged sword. The same inflammatory pathways that support tumor rejection may also increase toxicity, accelerate bacterial clearance, or contribute to systemic inflammatory complications [82, 91, 92]. Thus, the therapeutic value of bacteria-induced innate immunity depends not only on the strength of inflammatory activation, but also on its spatial control, duration, and safety profile.

### Priming of adaptive anti-tumor immunity

Beyond innate activation, bacterial therapies can also support adaptive anti-tumor immunity by promoting dendritic-cell maturation, cross-presentation of tumor antigens, and activation of tumor-specific cluster of differentiation 8-positive (CD8^+^) T cells. Some engineered bacterial platforms further amplify these effects by delivering tumor antigens, cytokines, or chemokines directly within the tumor microenvironment [39, 93–95]. In this context, bacteria may function not only as cytotoxic agents, but also as in situ vaccine-like systems.

The adaptive dimension is particularly important because durable tumor control generally requires more than transient local inflammation. Cytotoxic T lymphocytes can target residual malignant cells beyond the most heavily colonized regions, while memory responses may contribute to longer-term protection against recurrence. Accordingly, several bacterial platforms have been engineered to express cytokines such as interleukin-2 (IL-2), IL-12, and interleukin-18 (IL-18), or chemokines such as CC motif chemokine ligand 21 (CCL21), in order to recruit and activate T cells and antigen-presenting cells [39, 96–98].

However, the quality and durability of adaptive priming likely depend on several unresolved variables, including the extent of tumor-antigen release, the timing of bacterial clearance, the presence of local immunosuppressive cell populations, and whether the induced inflammatory milieu favors effective tumor rejection rather than immune exhaustion or tissue damage [10, 49, 51, 84, 99]. These issues remain insufficiently characterized in many preclinical studies, which often emphasize tumor regression without fully addressing the specificity, persistence, or long-term durability of the immune response.

### Remodeling of the tumor microenvironment

One of the most conceptually important roles of bacterial therapy is its ability to remodel the tumor microenvironment rather than merely serve as a delivery vehicle. Bacteria and their products may alter cytokine balance, reduce immune exclusion, recruit myeloid and lymphoid effector cells, affect extracellular matrix (ECM) organization, and, in some cases, reduce suppressive populations such as regulatory T (Tregs) cells [49, 51, 100]. These changes may render tumors more susceptible to radiotherapy, chemotherapy, and immune checkpoint blockade.

Bacteria can modify the immunosuppressive tumor microenvironment by increasing immune-cell infiltration, reducing regulatory T-cell abundance, and disrupting ECM barriers [38, 101]. Several studies suggest that certain bacterial systems can recruit neutrophils, macrophages, and natural killer cells to the tumor site, thereby helping to initiate or amplify local anti-tumor immunity [102–105]. Because Tregs cells suppress anti-tumor immune responses, bacterial strategies that reduce their abundance within the tumor microenvironment may help relieve local immunosuppression and permit a stronger immune attack [106, 107]. In parallel, the ECM can act as a structural barrier that impedes immune-cell access to malignant tissue. Bacteria or their products may modify this barrier and thereby facilitate immune infiltration [7]. Consistent with this concept, Zhang et al. showed that engineered bacteria capable of disrupting the tumor’s physical barrier significantly improved immune-cell infiltration and enhanced the efficacy of radio-immunotherapy in solid tumors [36].

This microenvironmental remodeling may also help explain why many apparently different bacteria-based combination strategies converge on similar outcomes. Although the platforms vary—including enzyme-prodrug systems, cytokine secretion, phototherapy coupling, radionuclide delivery, and checkpoint nanobody release—they often share common mechanistic endpoints: improved activity in hypoxic regions, greater local drug concentration, enhanced antigen release, increased immune-cell infiltration, and increased tumor-cell apoptosis [108–112].

Taken together, these foundational mechanisms show that bacterial-based cancer therapy is not defined by one dominant mode of action. Rather, its significance lies in the integration of targeting, cytotoxicity, immune activation, and microenvironmental remodeling. This multifunctionality helps explain both the promise of the field and the difficulty of translating it clinically, since each mechanistic advantage also introduces parallel challenges related to control, safety, and reproducibility.

## Therapeutic platforms and engineering strategies

**Fig. 3:**
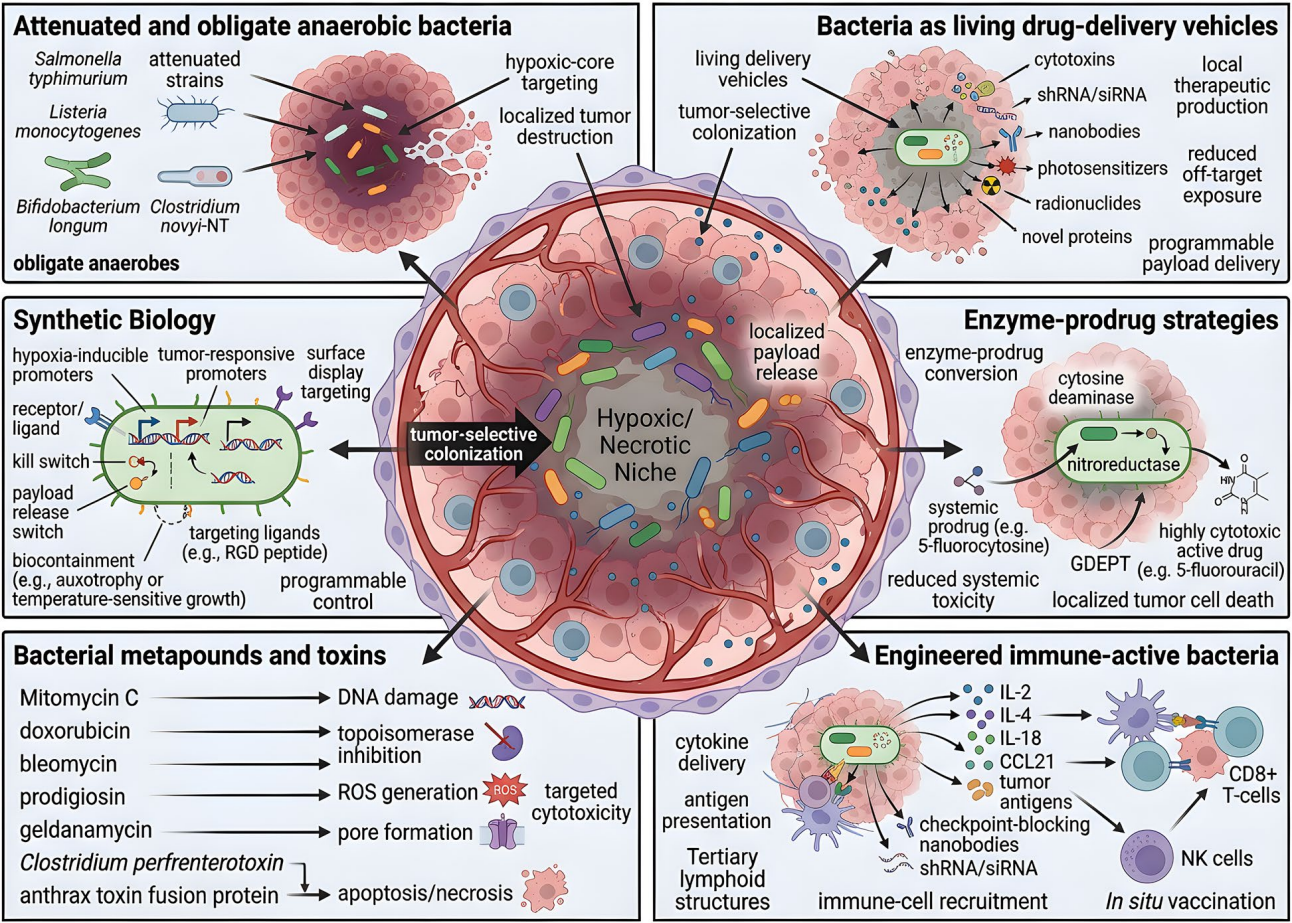
Therapeutic platforms and engineering strategies in bacterial-based cancer therapy. Bacteria-based cancer therapy includes multiple therapeutic platforms, ranging from attenuated or obligate anaerobic strains that selectively colonize hypoxic tumor regions to engineered systems capable of localized drug delivery, enzyme-prodrug conversion, immune activation, and precise payload control. Synthetic biology further expands these strategies through tumor-responsive promoters, surface-display targeting, and programmable safety mechanisms. Together, these approaches illustrate how natural bacterial tumor tropism can be integrated with engineering design to enhance selectivity, efficacy, and translational potential. Arginyl-Glycyl-Aspartic acid (RGD). Created in BioRender, https://BioRender.com

The therapeutic value of bacteria in oncology depends not only on their natural biology, but also on the engineering strategies used to shape their behavior, improve selectivity, and control toxicity. Current platforms can be broadly organized into several categories: attenuated or naturally tumor-tropic bacteria as monotherapies, bacteria as delivery vehicles, enzyme-prodrug systems, engineered immune-active strains, bacterial metabolites and toxins, and emerging synthetic-biology platforms with programmable control (Fig. 3, and Table 1).

**Table 1:** Bacterial therapeutic platforms, payloads, metabolites, toxins, and immune-modulating systems in cancer therapy

Category	Bacterial platform/ product	Payload/ active factor	Main mechanism	Therapeutic relevance	Key limitation	References
**Engineered platform**	*S. typhimurium*	Cytosine deaminase	Converts 5-FC to 5-FU within tumors	Local prodrug activation with reduced systemic toxicity	Requires reliable colonization and enzyme expression	[113, 118, 196, 197]
	*S. typhimurium*	shRNA/siRNA	Silences oncogenic or resistance-related pathways	Combines tumor targeting with molecular therapy	Cargo stability and delivery efficiency	[113]
	*S. typhimurium*	Therapeutic genes/payloads	Intratumoral delivery of anti-cancer molecules	Flexible bacterial chassis for tumor-targeted therapy	Biosafety and clearance control	[32, 66, 113, 118, 129, 130]
	*L. monocytogenes*	Tumor antigens/ immunomodulatory proteins	Stimulates antigen presentation and cytotoxic T-cell activation	In situ vaccine-like activity	Inflammatory toxicity and safety concerns	[89, 90, 156, 157]
	*E. coli*	Cytokine plasmids/ therapeutic proteins	Local release of immune-active or anti-cancer cargos	Programmable intratumoral delivery	Plasmid stability and expression control	[151–153, 155, 198]
	*B. longum*	Drug/gene cargos	Colonizes hypoxic tumor regions and delivers therapy locally	Strong selectivity for anaerobic tumor niches	More limited use outside hypoxic tumors	[73, 199]
	*C. novyi*-NT	Spores/lytic activity	Germinates in necrotic tumor cores and induces localized tissue destruction	Highly selective hypoxic-core targeting	Limited activity in oxygenated tumor regions	[38, 56, 200]
	Bacterial membrane vesicles/ engineered membranes	Ligands, proteins, antigens	Cell-free delivery of therapeutic or immune-active molecules	Reduced replication-associated risk	May lose self-amplifying colonization advantage	[120]
	Bacteria + photosensitizer	pDA/Ce6 or related agents	Bacterial targeting combined with light-activated tumor killing	Improved spatial control	Requires external activation	[119]
	Bacteria + nanotechnology	Nanoparticles/hybrid cargos	Combines bacterial tropism with controlled payload transport	Multifunctional and precise delivery	Greater manufacturing complexity	[193–195]
**Immune-modulating system**	*S. typhimurium*	IL-2	Promotes T-cell proliferation and NK-cell activation	Enhances anti-tumor immunity and tumor regression	Cytokine toxicity if poorly controlled	[147, 148]
	Engineered *E. coli*/ *Salmonella*	IL-4	Modulates local immune responses and may enhance cytotoxic activity in selected settings	Local immune remodeling	Context-dependent effects	[149–152]
	*Listeria*/*Salmonella*	IL-18	Stimulates IFN-γ production, Th1 polarization, dendritic-cell maturation, and NK-cell activation	Strengthens anti-tumor immune responses	Risk of excessive inflammation	[153–155]
	Engineered bacterial vectors	IFN-γ	Activates macrophages and cytotoxic immune pathways	Enhances local immune activation	Systemic inflammation if not contained	[13, 38, 101, 201–204]
	Engineered bacterial vectors	Interleukin-15 (IL-15)	Supports NK-cell and CD8^+^ T-cell survival and activation	Sustained local immunostimulation	Requires tight expression control	[13, 201–204]
	*S. typhimurium*	CCL21	Recruits dendritic cells and naïve T cells into tumors	Promotes immune-cell infiltration and local priming	Variable effect across tumor types	[146, 158, 159]
	*L. monocytogenes*/ *Salmonella*	Tumor antigens + cytokine support	Enhances antigen presentation and cytotoxic T-cell priming	In situ vaccine-like approach	Balance between efficacy and inflammation	[144, 145, 156,157, 205]
	Engineered bacteria	Anti-Programmed Death-Ligand 1 (PD-L1) nanobody	Local checkpoint blockade within tumor tissue	Converts poorly inflamed tumors into immune-active lesions	Payload stability and biosafety	[13, 201–204]
**Metabolite/toxin**	Mitomycin C	DNA cross-linking agent	Causes interstrand DNA cross-links	Established chemotherapeutic agent	Resistance linked to DNA repair pathways	[161, 162]
	Doxorubicin	Anthracycline	DNA intercalation, topoisomerase II inhibition, reactive oxygen species (ROS) generation	Widely used anti-cancer drug	Cardiotoxicity and systemic toxicity	[161, 163, 164]
	Bleomycin	DNA-cleaving antibiotic	Induces single- and double-strand DNA breaks via free radicals	Standard therapy in Hodgkin lymphoma and testicular cancer	Pulmonary and skin toxicity	[161, 165–167]
	Prodigiosin	Red-pigmented bacterial metabolite	Induces apoptosis and modulates cytokine signaling	Experimental anti-cancer and chemo-sensitizing activity	Limited translational validation	[168]
	Geldanamycin	Hsp90 inhibitor	Destabilizes oncogenic client proteins	Mechanistically important anti-cancer scaffold	Hepatotoxicity and formulation issues	[169]
**Enzyme therapeutic**	L-asparaginase	Asparagine-depleting enzyme	Starves leukemia cells of asparagine	Established therapy in acute lymphoblastic leukemia	Immunogenicity and resistance	[139, 140]
**Toxin**	*C. perfringens* enterotoxin	Claudin-3/−4-targeting toxin	Forms membrane pores in claudin-expressing tumor cells	Selective targeting of epithelial tumors	Off-target toxicity if targeting is incomplete	[170–172]
	Anthrax toxin fusion proteins	Receptor-directed toxin system	Selective cytotoxic delivery to tumor cells	Experimental targeting of receptor-positive tumors	Safety and delivery optimization needed	[173]
**Direct bacterial factor**	Multiple bacterial species	ROS/oncolytic factors	Oxidative stress and direct tumor-cell injury	Contributes to bacterial cytotoxicity	Difficult to control quantitatively	[7, 74–77]

*5-FC* 5-fluorocytosine, *5-FU* 5-fluorouracil, *shRNA* short hairpin RNA, *siRNA* small interfering RNA, *pDA/Ce6* polydopamine and chlorin e6, *IL* interleukin, *NK* natural killer, *IFN-γ* interferon-gamma, *CD8*^+^ Cluster of differentiation 8-positive, *CCL21* C–C motif chemokine ligand 21, *PD-L1* programmed death-ligand 1, *Hsp90*, heat shock protein 90, *ROS* reactive oxygen species

### Attenuated and obligate anaerobic bacteria as therapeutic monotherapies

Attenuated strains and obligate anaerobes represent the most historically recognizable form of bacterial cancer therapy. Their rationale is straightforward: to exploit the natural tumor tropism of bacteria while reducing pathogenicity sufficiently to permit therapeutic use. Representative examples include attenuated *S. typhimurium*, *L. monocytogenes*, *B. longum*, and *C. novyi*-NT.

These platforms are particularly relevant when the desired mechanism is direct intratumoral proliferation, hypoxic-core targeting, or localized tissue destruction. Attenuated *S. typhimurium* strains have been engineered to express short hairpin RNAs targeting oncogenes or enzymes such as cytosine deaminase, which locally converts prodrugs such as 5-fluorocytosine (5-FC) into cytotoxic compounds within tumors [113]. Obligate anaerobes such as *Clostridium* are especially well suited to necrotic and oxygen-deprived tumor compartments, whereas attenuated facultative strains may offer greater engineering flexibility and better compatibility with therapeutic payload delivery [114–117]. A clinical pilot study demonstrated this principle by using *Salmonella* expressing the *E. coli* cytosine deaminase gene, thereby enabling localized drug activation and reducing systemic toxicity [118].

More recent advances have integrated phototherapy and nanotechnology with attenuated bacterial platforms. For example, bacteria equipped with photosensitizers such as polydopamine and chlorin e6 (pDA/Ce6) have shown enhanced phototherapeutic efficacy with improved spatial control and reduced damage to surrounding tissue [119]. Similarly, bacterial membranes and vesicles have been engineered to carry tumor-targeting ligands, proteins, or antigens [120]. Nonetheless, the same features that support intratumoral persistence can also complicate dose control, clearance, and biosafety. Thus, although these monotherapy platforms provide important proof of concept for tumor-selective colonization, in current practice they often serve as chassis for further engineering rather than as unmodified living drugs.

### Bacteria as living drug-delivery vehicles

One of the most important advances in the field is the use of bacteria as active delivery vehicles rather than relying solely on their intrinsic cytotoxicity. This strategy addresses a major limitation of conventional oncology: many anti-cancer agents penetrate poorly into tumor cores or cause unacceptable systemic toxicity when administered broadly [12, 121–123]. By contrast, bacteria can localize to tumors and produce or release therapeutic cargo directly within diseased tissue [50, 123–125].

Bacterial payloads have included cytotoxic proteins, therapeutic genes, RNA interference constructs, radionuclides, nanobodies, photosensitizers, and immunomodulatory molecules [52, 93]. Living bacterial delivery systems such as *S. typhimurium*, *L. monocytogenes*, and *C. novyi* have been modified to selectively proliferate in hypoxic tumor environments, where they can induce cell death, deliver cytokines or apoptosis-inducing proteins, and stimulate host anti-tumor immunity [32, 66, 126–130]. This approach is attractive because it combines biological targeting with local production, potentially increasing intratumoral concentration while reducing off-target toxicity [34, 51]. It also allows bacterial therapy to be tailored toward distinct therapeutic goals, including direct tumor killing, stromal modulation, immune recruitment, and sensitization to partner treatments [37, 39, 131, 132].

### Targeted bacterial enzyme-prodrug strategies

Enzyme-prodrug systems are among the clearest examples of bacteria-mediated local pharmacology. In these approaches, tumor-colonizing bacteria express an enzyme that converts a systemically administered, relatively non-toxic prodrug into an active anti-cancer compound within the tumor. The classic example is cytosine deaminase-mediated conversion of 5-FC to 5-fluorouracil (5-FU) [133].

A related strategy is gene-directed enzyme prodrug therapy (GDEPT), in which a transgene encodes an enzyme capable of converting a prodrug into an active therapeutic metabolite. GDEPT relies on three components: the prodrug, an enzyme-encoding gene, and a delivery carrier. This selective intratumoral conversion is intended to confine toxicity to tumor tissue while minimizing exposure of healthy cells. One representative example involves bacterial nitroreductase, which can activate non-toxic compounds into cytotoxic drugs specifically at the tumor site. Emerging studies suggest that such approaches may reduce systemic toxicity while improving tumor control [134–136].

This strategy remains appealing for several reasons. It creates a mechanistic separation between systemic exposure and local activation, exploits bacterial localization without requiring bacteria themselves to be the primary cytotoxic agents, and can in principle be adapted to multiple enzyme-prodrug pairs. However, its success depends on reliable tumor colonization, sufficient local enzyme expression, and limited off-target bacterial persistence. Major translational challenges therefore include variability in colonization density, uneven enzyme expression, and the possibility that ectopic bacterial survival could generate unintended local toxicity [42, 137, 138].

Bacterial enzymes can also function as anti-cancer therapeutics outside the classic prodrug framework. A prominent example is L-asparaginase, derived from *E. coli* or *Erwinia*, which depletes asparagine and is widely used in leukemia treatment. Because leukemia cells are often unable to synthesize sufficient asparagine, depletion of this amino acid can induce cell death. This strategy has contributed to survival rates approaching 90% in pediatric acute lymphoblastic leukemia [139]. Despite its success, immunogenicity and resistance remain concerns, motivating the development of improved variants and engineered alternatives [140]. Overall, enzyme-based strategies remain among the most clinically interpretable bacteria-derived therapies because their logic closely parallels established pharmacologic principles.

### Engineered bacteria expressing cytokines, antigens, or gene-regulatory cargos

Engineered bacteria can also be designed to deliver immunostimulatory cytokines, chemokines, tumor-associated antigens, apoptosis-inducing molecules, checkpoint-blocking nanobodies, and regulatory cargos such as short hairpin (shRNA) or small interfering RNA (siRNA) [56, 141–145]. Upon colonization of tumor tissues, these bacterial vectors can stimulate innate and adaptive immune pathways through interaction with pattern-recognition receptors on antigen-presenting cells, thereby triggering downstream cytokine cascades [146].

Several cytokines have been explored in this context. IL-2 is central to T-cell proliferation and natural killer cell activation, and bacterial delivery of IL-2 has been associated with tumor regression in preclinical models [147, 148]. IL-4, although classically associated with Th2 responses, may also contribute to anti-tumor effects under selected immunologic conditions, particularly when co-expressed with IL-2 or IL-18 [149, 150]. Engineered *E. coli* and *S. typhimurium* have been modified to express IL-4 under tumor-specific or hypoxia-inducible promoters, enabling localized cytokine delivery while limiting systemic toxicity [151, 152]. IL-18, which promotes IFN-γ production and Th1 polarization, has likewise shown promise when delivered through bacterial vectors, leading to dendritic-cell maturation, enhanced natural killer (NK)-cell activity, and tumor necrosis in experimental models [153–155].

In addition to cytokines, bacterial systems have been engineered to express tumor antigens or immunomodulatory proteins directly within the tumor microenvironment. For example, *L. monocytogenes* and *Salmonella* have been designed to secrete tumor antigens or immune-active proteins that stimulate cytotoxic T-cell responses [156, 157]. Chemokine CCL21 is also of particular interest because it recruits naïve T cells and dendritic cells into tumors [146, 158]. Notably, *S. typhimurium* engineered to express CCL21 has been reported to remodel the tumor milieu and promote the formation of tertiary lymphoid structures that support local immune priming and tumor rejection [159]. Collectively, these platforms highlight how engineered bacteria can act not only as direct anti-tumor agents, but also as in situ immune modulators and vaccine-like systems, particularly in solid tumors that respond poorly to conventional checkpoint blockade [160].

### Bacterial metabolites and toxins as anti-cancer agents

Bacteria can also produce a wide range of bioactive compounds, including metabolites, toxins, and enzymes, that have anti-cancer activity through distinct mechanisms. Some bacterial secondary metabolites, such as mitomycin C, doxorubicin, and bleomycin, have already entered clinical oncology as the US Food and Drug Administration (FDA)-approved chemotherapeutic agents [161]. Mitomycin C acts primarily through DNA cross-linking, and its activity has been linked to interstrand cross-link formation, while resistance is influenced in part by DNA-repair pathways such as the Fanconi anemia/homologous recombination system [162, 163]. Anthracyclines such as doxorubicin, originally derived from *Streptomyces*, intercalate into DNA and inhibit topoisomerase II, thereby inducing DNA damage and reactive oxygen species. Newer ATPase-site inhibitors such as topobexin are being explored in an effort to reduce anthracycline-associated toxicity while preserving anti-tumor potency [164].

Bleomycin represents another important bacterial-derived anti-cancer agent. It binds GC-rich DNA regions and, through an iron-oxygen complex, generates free radicals that induce single- and double-strand DNA breaks [165, 166]. This mechanism contributes to its continued clinical use in Hodgkin lymphoma and testicular cancer. However, bleomycin toxicity, particularly in the lungs and skin, is linked to low levels of the protective enzyme bleomycin hydrolase, which helps explain its tissue-specific adverse effects [167].

Other bacterial products with emerging anti-cancer potential include prodigiosin, a red-pigmented metabolite from *Serratia marcescens* that has apoptosis-inducing and immunomodulatory activity, and geldanamycin from *S. hygroscopicus*, which targets heat shock protein 90 (Hsp90) and destabilizes oncogenic proteins [168, 169]. Although these compounds show considerable promise, toxicity and delivery remain important barriers to clinical translation.

Beyond metabolites, bacterial toxins may provide additional targeted anti-cancer tools. *C. perfringens* enterotoxin (CPE) binds claudin-3 and claudin-4, which are often overexpressed in epithelial tumors, and exerts rapid pore-forming cytotoxicity [170]. Preclinical studies have shown that CPE-based gene therapy can eradicate colon carcinoma xenografts within days [171]. Delivery systems such as claudin-targeted nanoparticles have been developed to improve tumor specificity and reduce off-target effects [172]. More recently, anthrax toxin fusion proteins have demonstrated selective killing of human epidermal growth factor receptor 2 (HER2)-positive tumor cells while sparing normal tissue [173].

An important advantage of metabolite- and toxin-based strategies is that they partially decouple therapeutic efficacy from live bacterial burden. In principle, toxins, peptides, metabolites, or outer-membrane vesicles may preserve anti-tumor activity while reducing some of the risks associated with uncontrolled bacterial replication. However, they may also sacrifice one of the principal strengths of live bacterial systems: self-amplifying colonization within otherwise inaccessible tumor compartments. For this reason, bacterial metabolites and toxins may be especially useful when integrated into controlled delivery platforms rather than viewed as simple substitutes for live bacteriotherapy.

### Synthetic biology and precision engineering

Recent advances in synthetic biology and genetic engineering have enabled the development of more sophisticated bacterial systems capable of selectively targeting and eradicating tumor tissue. These engineered bacteria can be designed to express receptor genes [174, 175], cytotoxic proteins [176, 177], anti-cancer molecules [40, 178], and tumor-associated antigens [45, 179], thereby improving tumor specificity and therapeutic precision.

Bacterial vectors such as *Salmonella*, *Clostridium*, *Bifidobacterium*, and *Listeria* naturally colonize hypoxic tumor microenvironments [127, 180–182], and this property has been harnessed to deliver therapeutic payloads such as tumor suppressor proteins, cytokines, and death receptor ligands directly to tumor tissue [11]. For example, engineered strains have been developed to express cytotoxic proteins such as azurin, which can selectively induce apoptosis in cancer cells [67, 69, 183, 184]. Other strategies use bacterial surface-display systems to present ligands or receptors that improve tumor-marker recognition and selective accumulation [184–187]. In addition, tumor-responsive promoters, including arabinose-inducible (pBAD) and hypoxia-inducible systems, have been employed to restrict payload release to the tumor microenvironment and thereby reduce systemic toxicity [157, 188–192].

An especially promising direction is the integration of engineered bacteria with nanotechnology. Such systems can deliver not only cytotoxic proteins, but also chemotherapeutics, immune modulators, or gene-editing components directly into cancer cells [193]. These multifunctional platforms offer the possibility of combining targeted killing with immune activation, including stimulation of NK cells and CD4^+^ T cells [194, 195]. Overall, these strategies represent a next-generation paradigm in bacterial-based cancer therapy, one that seeks to combine biological targeting with programmable control and precision payload delivery.

## Combination strategies in the context of tumor biology and therapeutic mechanism

**Fig. 4:**
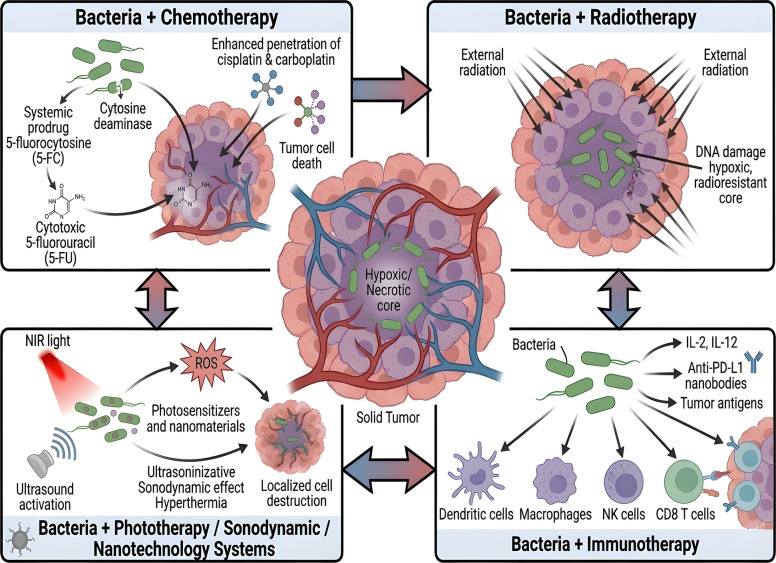
Combination strategies in bacterial-based cancer therapy. Tumor-targeting bacteria can enhance conventional and emerging cancer therapies through complementary mechanisms. In chemotherapy combinations, bacteria improve local drug delivery or activate prodrugs within tumors. In radiotherapy combinations, bacteria preferentially target hypoxic and radioresistant tumor regions. In phototherapy, sonodynamic, and nanotechnology-enabled systems, bacteria function as biological navigators for triggerable payload delivery. In immunotherapy combinations, bacteria deliver cytokines, nanobodies, or tumor antigens and promote local immune activation. Together, these exploit bacterial strategies tumor tropism to improve treatment precision and therapeutic synergy. Created in BioRender, https://BioRender.com

Combination strategies are most persuasive when interpreted through tumor biology rather than as a simple list of partner modalities. Bacterial platforms are not universally superior across all tumors; instead, their relative value depends on which tumor constraints dominate in a given setting, such as hypoxia and necrosis, poor drug penetration, immune exclusion, stromal barriers, or acquired resistance [10, 31, 51, 53, 85, 206, 207]. From this perspective, chemotherapy combinations are often most useful when bacteria improve intratumoral drug activation or concentration, radiotherapy combinations are particularly relevant for hypoxic and radioresistant regions, and immunotherapy combinations are most compelling when bacteria can convert immunologically “cold” tumors into inflamed lesions with greater antigen presentation and effector-cell recruitment [38, 178] (Fig. 4 and Table 2). Viewing these strategies through shared mechanistic logic also clarifies why apparently different platforms frequently converge on similar benefits, including improved apoptosis, better activity in poorly perfused tumor regions, enhanced immune infiltration, and greater local therapeutic intensity. This framework helps distinguish when a bacterial strategy functions mainly as a delivery system, when it acts as an immune modulator, and when it serves as a multifunctional partner in rational combination therapy [60].

**Table 2:** Synergistic interactions between bacteriotherapy and conventional cancer modalities

Name	Therapy type	Cancer type	Study model	Mechanism of action	Outcomes	References
*S. typhimurium*	Bacteriotherapy (intratumoral injection)	Human prostate cancer (PC-3)	In vivo: nude mice	Induction of apoptosis	Complete tumor regression with no observable toxicity	[62]
*L. rhamnosus* GG	Bacteriotherapy and chemotherapy	Colon and metastatic melanoma	In vitro: human colon cancer cell lines	Sensitizes cancer cells to both 5-FU and irinotecan	Positive synergistic action	[219]
*L. monocytogenes*-based vaccine	Bacteriotherapy and radiotherapy	Melanoma (B16-OVA)	In vivo: C57BL/6 mice	*Listeria* promotes tumor antigen presentation and T-cell activation, radiation therapy increases tumor immunogenicity	Significant tumor regression and prolonged survival	[220]
*L. monocytogenes* (Lm^at^ -LLO)	Bacteriotherapy and radiotherapy	Melanoma	Mice (Braf/Pten)	Lm^at^-LLO induced ROS and apoptosis in tumor cells, and enhances immune response within tumor	Reduced tumors and metastases	[221]
*L. rhamnosus* GG and 5-FU	Bacteriotherapy and chemotherapy	Colon cancer (Caco-2)	Caco-2 human carcinoma cells	Pro-inflammatory cytokine upregulation	Enhances cytokine response; safer immune modulating benefits	[222]
*B. longum* engineered with cytosine deaminase	Bacteriotherapy and chemotherapy	Colorectal (CT26)	CT26 tumor bearing mice	Local 5-FC to 5-FU conversion	Prolonged survival, enhanced efficacy over monotherapy	[223]
*C. novyi*-NT spores	Bacteriotherapy and chemotherapy	Cervical cancer (TC-1)	In vivo: TC-1 cell line in C57BL/6 mice	Spore germination triggers ROS-mediated apoptosis and ceramide increase via mitochondrial pathway	Significant tumor regression and volume reduction	[224]
*E. coli* K-12 and radiation	Bacteriotherapy and radiotherapy	Colon carcinoma (CT26)	BALB/c mice with syngeneic tumors	Bacteria lyse hypoxic tumor regions using Cytolysin A (ClyA); radiation therapy kills normoxic proliferative cells	Produced significant shrinkage and removal of CT26 tumors in mice, also suppressed lung metastases and prolonged survival	[225]
*S. typhimurium* and radiation	Bacteriotherapy and radiotherapy	Colon carcinoma (CT26)	BALB/c mice with CT26 tumors	Bacterial cytolysis in hypoxic regions enhances therapy effect in conjunction with radiotherapy	Improved tumor control	[226]
*C. novyi*-NT and radiation	Bacteriotherapy and radiotherapy (radio-immunotherapy, brachytherapy)	Colon (HCT116, HT29), Cholangiocarcinoma (HuCCT-1), Melanoma (B16)	Mice (transplanted xenografts)	Anaerobic germination destroys hypoxic, radio resistant tumor cores; sensitizes tumor to Radiotherapy	Improved efficacy of radiation therapy	[227]
*L. monocytogene*s	Bacteriotherapy and radiotherapy (targeted radionuclide)	Metastatic pancreatic cancer (Panc-02)	In vivo: mouse Panc-02 metastasis model	*Listeria* accumulates in metastases and delivers β(B) radiation to kill tumor cells while not harming the normal tissue	Multiple low dose treatments reduced metastases by ~ 90%	[228]
*B. infantis* and 5-FC	Bacteriotherapy and chemotherapy (prodrug activation)	Melanoma (B16-F10)	In vitro: B16-F10 melanoma cells In vivo: mice with B16-F10 xenograft	Bacterial cytosine deaminase converts gene 5-FC to 5-FU within tumors	Combination inhibited tumor volume vs controls	[196]
*B. longum* and 5-FC	Bacteriotherapy and chemotherapy (prodrug activation)	Autochthonous mammary tumors	Rats with 7,12-dimethylbenz[a]anthracene induced mammary tumors	Tumor localized cytosine deaminase converts 5-FC to 5-FU at site, increases intratumoral 5-FU	Demonstrated antitumor efficacy with tumor volume reduction after intratumoral/IV delivery alongside 5-FC	[229]
*L. acidophilus*, *L. casei* (live or irradiated) and 5-FU	Bacteriotherapy and chemotherapy	Colorectal cancer (LS513 cells)	In vitro: human LS513 colorectal cancer cell line	Probiotic lactic acid bacteria increased 5- FU induced apoptosis, faster caspase −3 activation, p21 downregulation	An increase in apoptotic efficacy compared to 5-FU alone	[58]
*S. typhi* and cisplatin	Bacteriotherapy and chemotherapy	Ovarian cancer (cisplatin resistant SKOV-3/DDP)	In vivo: BALB/c nude mice with SKOV-3/DDP xenografts	Bacterial delivery of multidrug resistance protein 1 siRNA downregulates P-glycoprotein reversing cisplatin resistance, therefore reducing efflux and restoring drug sensitivity	Tumors grew slower and were more sensitive to cisplatin vs. control	[230]

*5-FC* 5-fluorocytosine, *5-FU* 5-fluorouracil

### Synergistic combinations of bacteriotherapy and chemotherapy

The combination of bacteriotherapy and chemotherapy is especially attractive for inoperable or treatment-resistant cancers because it may enhance anti-tumor efficacy while reducing systemic toxicity [60]. Conventional chemotherapeutic agents are often limited by inadequate intratumoral drug concentration, off-target cytotoxicity, and acquired resistance [38, 53]. In contrast, genetically engineered bacteria can selectively target hypoxic and necrotic regions that are poorly reached by standard drugs [56].

Bacteria can also function as delivery vehicles for chemotherapeutic agents or for enzymes that convert non-toxic prodrugs into active cytotoxic compounds directly within the tumor [38]. A well-known example is cytosine deaminase-mediated conversion of 5-FC to 5-FU at the tumor site [196, 197]. This localized drug activation increases intratumoral drug exposure while limiting systemic toxicity. Additional studies have shown that combining *S. choleraesuis* with cisplatin can delay tumor progression and prolong survival in preclinical models [208]. Other strategies, such as focused ultrasound ablation combined with *Bifidobacterium* and carboplatin, have also been explored to improve tumor-specific drug delivery [199]. Overall, the value of bacteria-chemotherapy combinations lies in spatial complementarity: bacteria preferentially localize to poorly perfused tumor compartments, whereas chemotherapy is often less effective in those regions.

### Bacterial targeting of radioresistant hypoxic niches

Combining bacteriotherapy with radiotherapy offers a rational strategy for improving local tumor control, especially in hypoxic tumors [38, 53]. Solid tumors often contain poorly oxygenated areas that are resistant to radiation, since ionizing radiation is most effective when sufficient oxygen is present in the targeted tissue [53, 209]. This limitation creates an opportunity for bacteria, which can preferentially colonize these oxygen-deprived regions [62].

Facultative or obligate anaerobes such as *S. typhimurium* and *C. novyi*-NT can proliferate in hypoxic and necrotic tumor areas that are largely absent from healthy tissues [38, 200]. This creates a mechanistic division of labor: radiotherapy targets better-oxygenated tumor regions, whereas bacteriotherapy acts within hypoxic compartments [60]. Engineered *S. typhimurium* strains carrying therapeutic payloads have shown stronger tumor suppression when combined with radiotherapy than with bacterial therapy alone [181]. In one colon tumor model, radiotherapy increased *S. typhimurium* colonization, and the combined treatment significantly inhibited tumor growth [181]. These findings suggest that the synergy between bacteriotherapy and radiation reflects not only additive cytotoxicity, but also complementary targeting of distinct tumor niches.

### Phototherapy, sonodynamic, and nanotechnology-enabled combinations

Emerging combinations involving photothermal therapy, photodynamic therapy, sonodynamic activation, focused ultrasound, and nanoparticle-assisted delivery reflect the growing integration of bacterial therapy with engineering-based oncology platforms. In these systems, bacteria act as biological navigators that carry photosensitizers, nanomaterials, or triggerable payloads into tumors, where therapy can then be spatially activated [210–214]. For example, engineered *E. coli* expressing photosensitizers has achieved highly selective tumor phototherapy [119].

The main advantage of these strategies is precision. External activation by ultrasound or light retains the targeting advantage of bacteria while improving temporal control over payload release, thereby reducing constitutive expression and limiting activity to the tumor site [214]. At the same time, these multimodal systems add complexity in design, equipment requirements, and reproducibility [211, 215]. Thus, although they are highly promising in proof-of-concept studies, their clinical translation may be more challenging.

### Modulating the tumor microenvironment via bacterial immunotherapy

Bacteria have emerged as promising tools for delivering immunotherapeutic agents directly to tumors because of their natural tropism for hypoxic, necrotic, and immune-privileged tumor cores [11, 126, 216]. Their selective localization enables local release of cytokines, immune checkpoint inhibitors, nanobodies, and antigenic payloads, potentially enhancing anti-tumor immunity while reducing systemic toxicity [53, 54, 62, 178, 207, 217, 218]. In addition to acting as delivery vehicles, bacterial colonization itself can stimulate anti-tumor immune responses and modulate the tumor microenvironment in ways that may improve the efficacy of immune checkpoint blockade [53, 57, 60].

A clinically established example is *Bacillus Calmette-Guérin* (BCG), an attenuated strain of *Mycobacterium bovis* approved for non-muscle-invasive bladder cancer, where it reduces recurrence and progression [23, 53, 62]. Other approaches remain largely preclinical but are mechanistically promising. For example, attenuated *S. typhimurium* has been used as a vector for a legumain-based minigene vaccine designed to suppress tumor-associated macrophages. Additional engineered bacterial systems have been designed to express IL-2, IFN-γ, IL-15, or anti-PD-L1 nanobodies in response to tumor microenvironmental cues [13, 201–204]. These constructs can stimulate tumor-infiltrating lymphocytes, amplify local immune activation, and in some cases label tumors for attack by chimeric antigen receptor-modified immune cells [136–139]. One clinical demonstration involved *E. coli* engineered to carry plasmids encoding immunotherapeutic proteins, resulting in tumor-specific accumulation and localized cytokine expression after intravenous administration [198]. Together, these findings suggest that bacteria-based immunotherapy may function as a localized and programmable strategy to convert poorly inflamed tumors into more immunologically active lesions.

## Translational progress and clinical landscape

Despite strong preclinical momentum, the central question for bacterial-based cancer therapy is no longer whether bacteria can suppress tumors under experimental conditions, but whether they can do so safely, reproducibly, and controllably in human patients. This translational perspective is essential because one of the major criticisms of the original manuscript was that it emphasized therapeutic promise without adequately examining the barriers that separate preclinical success from clinical reality.

### Clinical precedents and early-phase experiences

A defining feature of bacterial-based oncology therapeutics is the tension between tumor-local efficacy and systemic safety. Early human experiences show that attenuation can enable administration but does not guarantee objective tumor responses, highlighting the need for improved localization, controllable payload release, and rational combinations. In a phase I study of IV VNP20009 (attenuated *S. typhimurium*) in metastatic melanoma, some focal tumor colonization was observed at higher doses, but no objective tumor regression occurred; dose-limiting toxicities included hematologic and hepatobiliary signals and persistent bacteremia in certain dosing cohorts [231–233]. These results are consistent with the field-wide shift from relying on colonization alone toward engineering strategies that (i) tighten tumor specificity, (ii) minimize systemic exposure, and (iii) engage immune pathways in a controlled manner.

Intratumoral delivery can increase local control but surfaces distinct safety constraints. In the first-in-human intratumoral *C. novyi*-NT spore study in advanced solid tumors, bacterial germination and lysis of injected tumor masses were observed in a subset of patients, but severe toxicities (including grade 4 sepsis and gas gangrene in specific contexts) underscored the importance of tumor-size selection, monitoring, and containment strategies [234, 235].

A modern engineered paradigm is illustrated by the probiotic *E. coli* Nissle platform engineered to produce cyclic dinucleotides under hypoxia to activate stimulator of interferon genes (STING) in phagocytic antigen-presenting cells within tumors (SYNB1891) [236, 237]. Early-phase clinical evaluation emphasizes local immune engagement and controllability rather than uncontrolled proliferation, providing a template for “mechanism-first” bacterial immunotherapy design [124, 236].

### Safety, host clearance, and genetic stability

Safety risks include bacteremia/sepsis, off-target colonization, cytokine-mediated toxicity, and unpredictable within-tumor distribution [49, 93, 98, 238]. Clinical data illustrate that both systemic delivery (risking bacteremia and systemic inflammation) and intratumoral injection (risking localized necrosis/infection-like complications) require stringent protocols and engineered containment [10, 12, 42, 93, 234]. A practical translational framework is to treat “biocontainment” as a first-class design constraint, using attenuation, auxotrophy, inducible lysis/kill-switches, antibiotic sensitivity, and (where possible) genomic integration for stability [239, 240].

Route of administration is also a major determinant of safety and should be treated as a design variable rather than a purely procedural choice. Intravenous delivery may be attractive for disseminated disease, but it increases exposure to systemic clearance mechanisms and raises the risk of transient bacteremia, inflammatory toxicity, and non-tumor biodistribution [121, 241]. By contrast, intratumoral or locoregional administration may improve local concentration and reduce systemic exposure, yet it introduces different constraints related to injection-site necrosis, local abscess-like reactions, tumor accessibility, and procedural reproducibility [242, 243]. These considerations are especially important in patients with advanced cancer, whose immune status, prior antibiotic exposure, mucosal barrier integrity, implanted devices, and treatment history may all influence bacterial persistence and safety. For this reason, translational development should not evaluate bacterial strains in isolation, but rather as integrated systems in which chassis, route, dose, persistence kinetics, and rescue strategy are co-optimized [37, 244]. In practical terms, a clinically viable bacterial platform should ideally have predictable colonization behavior, measurable clearance kinetics, retained sensitivity to clinically usable antibiotics, and a defined operational plan for containment, monitoring, and intervention if dissemination occurs [54, 215].

### Microbiome–disease databases and computational resources for bacterial-based oncology

Microbiome databases and computational tools are becoming increasingly useful in bacterial-based oncology because they help identify cancer-associated microbes, host–microbe interactions, and microbiota-related factors that may influence treatment response. Although most of these resources were not specifically developed for live bacterial therapeutics, they can support strain selection, biomarker discovery, and hypothesis generation for bacterial cancer therapy [245–247].

Several platforms are especially relevant. MicroPhenoDB curates associations among microbes, microbial genes, and disease phenotypes, whereas MicrobiomeNet integrates microbial associations with metabolic modeling [245, 246]. mBodyMap catalogs microbes across body sites and diseases, and Microbiomics of Anticancer Drug Efficacy and Toxicity (MADET) focuses on microbiota influences on anticancer drug efficacy and toxicity [248, 249]. More oncology-oriented resources are also emerging, including HGMT, which links gut microbiome features with tumor diagnosis and immunotherapy prognosis [250]. In addition, GutBalance and gutMEGA support microbiome-based biomarker discovery and cross-study comparison of disease-associated microbial patterns [247, 251].

These resources may contribute to bacterial-based oncology in three main ways: by helping identify candidate bacterial strains, by clarifying potential mechanisms through genes, metabolites, and pathways, and by supporting biomarker development for treatment response or toxicity [252]. However, most currently available data remain heterogeneous and largely correlative, and these platforms do not fully capture therapeutic variables such as genetic stability, payload control, biodistribution, or biocontainment. Therefore, microbiome databases should be viewed primarily as tools for hypothesis generation and translational prioritization rather than stand-alone guides for therapeutic design.

Another translational issue that deserves greater emphasis is the mismatch between common preclinical endpoints and the criteria that actually govern clinical development. Many animal studies still prioritize tumor-volume reduction over questions that are decisive in human translation, such as intratumoral distribution, persistence kinetics, systemic shedding, reversibility with antibiotics, manufacturability, compatibility with standard-of-care regimens, and the reproducibility of engineered function across batches and hosts [253, 254]. For bacterial therapeutics, these issues are particularly important because efficacy and safety are inseparable from biodistribution and controllability. A platform that appears highly active in a permissive mouse model may fail clinically if colonization is inconsistent, if engineered circuits lose function under physiologic stress, or if rescue measures are not reliable in the event of dissemination [254, 255]. Future translational studies should therefore be designed not only to show that a bacterial therapy can work, but to define under what tumor conditions it works, how its activity can be monitored in real time, and what operational parameters would support regulatory development and combination use in patients [256, 257].

## Conclusion and prospects

Bacterial-based cancer therapy has evolved from early observations of infection-associated tumor regression into a mechanistically diverse and increasingly engineerable therapeutic field. A central conclusion from the current literature is that bacteria should not be considered as a single anticancer modality, but rather as programmable biological platforms capable of integrating tumor targeting, direct cytotoxicity, localized drug delivery, immune activation, and remodeling of the tumor microenvironment. Their greatest promise lies in tumor settings that are poorly perfused, hypoxic, necrotic, immune-excluded, or otherwise difficult to treat using conventional therapies alone.

An important conclusion of this review is that the value of any bacterial strategy depends strongly on tumor characteristics and therapeutic context. Direct colonization or lytic approaches are most attractive in tumors dominated by hypoxic or necrotic niches. Engineered delivery systems are particularly advantageous when the major limitation is inadequate local drug exposure. Immune-active bacterial platforms appear especially promising in immunologically “cold” tumors, where innate immune activation, antigen release, and effector-cell recruitment may improve responsiveness to checkpoint blockade and other immunotherapies. structured, the field is most informative not when it merely presents isolated examples of efficacy, but when it clarifies how specific bacterial mechanisms match specific tumor constraints. More explicit alignment between biology and bacterial design is therefore essential for both conceptual clarity and tumor translation progress.

Despite this promise, several major barriers continue to limit clinical translation. One of the most important is tumor heterogeneity. Tumors differ widely in oxygenation, vascular permeability, stromal density, extent of necrosis, immune composition, metabolic state, and spatial organization [258, 259]. Even within a single lesion, different regions may vary considerably in their ability to support bacterial colonization or payload activity [33, 260]. Consequently, bacterial targeting is unlikely to be uniform across patients, tumor types, or even within different areas of the same tumor. This issue is particularly relevant for therapies that rely on hypoxic or necrotic targeting. Although such niches are common in many solid tumors, they are not equally accessible or biologically stable in all cases [261, 262]. As a result, strategies that perform well in tumors with large anaerobic cores may not translate as effectively to lesions with limited necrosis or more active immune surveillance [4, 262].

A central biological tension in bacterial therapy is the need to balance persistence with controllability [98, 215]. Bacteria must remain present long enough to colonize tumors, release payloads, activate immunity, or convert prodrugs. However, prolonged persistence may increase the risk of toxicity, dissemination, or unpredictable host responses. Host immune clearance adds another level of complexity: rapid clearance may reduce efficacy, whereas insufficient clearance may compromise safety [10, 41, 42, 98, 215]. This is not merely a technical consideration, but a core determination of treatment design. The therapeutic value of a bacterial platform depends on whether its persistence matches its intended mechanism of action. A short-lived bacterial burst may be sufficient for inflammatory priming or localized payload release, whereas sustained colonization may be required for enzyme–prodrug conversion or prolonged remodeling of the tumor microenvironment [9, 93].

Safety remains the single greatest obstacle to wider clinical application. Potential risks include bacteremia, sepsis, systemic inflammatory responses, colonization of non-tumor tissues, and adverse interactions in immunocompromised patients or in the presence of disgusting mucosal barriers and implanted medical devices [263, 264]. These risks are especially important in oncology, where patients may already have altered immune status as a result of prior therapy, advanced disease, or comorbidities. Importantly, attenuation alone is not a complete solution. A strain weakened enough to reduce pathogenicity may also lose therapeutic fitness, whereas a strain that retains strong colonization capacity may raise greater biosafety concerns [11, 51, 93, 124]. This trade-off helps explain why recent advances increasingly emphasize controllability rather than attenuation alone. Strategies such as auxotrophy, retained antibiotic sensitivity, inducible expression systems, and kill switches are therefore not optional refinements, but essential prerequisites for clinical viability [92].

As bacterial therapy becomes more sophisticated, the reliability of engineered function also becomes increasingly important. Genetic circuits, plasmid-borne payloads, inducible promoters, and multicomponent synthetic systems may impose significant metabolic burden or selective pressure on the bacterial host [265–267]. Over time, these systems may lose stability, mutate, show variable expression, or collapse altogether. Such issues are often underemphasized in proof-of-concept studies, yet they are highly relevant for clinical translation. This challenge is especially important for bacteria engineered to perform multiple functions simultaneously [268]. Although multifunctionality is conceptually attractive, it may reduce robustness in vivo. In many cases, the most translationally promising constructs may be those with a smaller number of tightly controlled functions rather than highly elaborate systems that are difficult to maintain consistently under physiological stress [265–267, 269].

Another under-discussed issue is the ecological behavior of bacteria after administration. Bacteria are not internal carriers; they can evolve, adapt, interact with local microbial communities, and form structured states such as biofilms [263, 270, 271]. In a therapeutic context, these properties raise important questions regarding persistence, containment, immune evasion, and responsiveness to antibiotics [46, 263]. The possibility of escape mutants or unintentional colonization patterns is particularly relevant for live engineered strains intended for systemic delivery. Future studies should therefore include more systematic analysis of bacterial fate after treatment, including tissue persistence, routes of clearance, genetic stability, and susceptibility to rescue interventions.

Although animal models remain essential for preclinical development, many do not adequately reproduce the immune, stromal, microbial, and treatment-history complexity of human cancer. This creates a significant risk of overinterpreting strong efficacy signals from simplified systems. In particular, interactions between therapeutic bacteria and the human microbiome, prior antibiotic exposure, inhibitor checkpoint history, and underlying immune heterogeneity remain insufficiently modeled [272, 273]. To improve translational relevance, future preclinical studies should incorporate more realistic designs, including immunocompetent models, spatially complex tumors, biodistribution analyses, antibiotic rescue studies, and longer follow-up to assess persistence, recurrence, and delayed toxicity [35, 274]. Progress in this field will depend not only on continued innovation in bacterial engineering, but also on more demanding translational benchmarks.

These limitations also help explain why preclinical findings are often more consistent than clinical outcomes. Many animal models do not capture the safety constraints, stromal architecture, immune complexity, and treatment history encountered in human cancer. A rigorous field therefore requires not only stronger efficacy claims, but also more explicit discussion of controllability, reproducibility, and failure modes.

Several priorities emerge from this synthesis. First, future bacterial therapeutics should incorporate tighter spatiotemporal control through tumor-responsive expression systems, kill switches, and improved biocontainment circuits. Second, platform selection should be guided more clearly by tumor biology, with clearer criteria for when to prioritize colonization-based killing, drug-delivery approaches, or immune reprogramming. Third, translational studies require better potency assays, more informative biomarkers of localization and immune activation, and preclinical models that better capture tumor heterogeneity and host–microbe interactions. Finally, microbiome–disease databases and computational resources may help refine strain selection, generate mechanistic hypotheses, and support biomarker-driven patient stratification.

A further priority for the field is biomarker-guided patient and tumor selection. Not all cancers are equally suitable for bacterial therapy, and one major reason for variable translational performance is likely the absence of clear selection criteria [34, 275]. Future work should move toward defining which measurable tumor features most strongly predict benefit, including the extent and spatial stability of hypoxia or necrosis, vascular permeability, stromal exclusion, baseline immune infiltration, and prior treatment history [34, 276]. The same principle applies to therapeutic design. Tumors dominated by inaccessible hypoxic cores may favor colonization-based or enzyme–prodrug strategies, whereas immune-excluded tumors may be better matched to cytokine-secreting or checkpoint-modulating bacterial systems [99, 277]. This type of framework is essential if bacterial therapy is to mature from an intriguing platform into a precision-guided treatment modality. Without explicit linkage between tumor phenotype and bacterial mechanism, even promising results will remain difficult to generalize across tumor types, study systems, and clinical settings [54, 60, 278].

Equally important is the need for development standards that treat engineered bacteria not merely as creative biologic tools, but as regulated therapeutic products. Clinical translation will depend on robust manufacturing workflows, strain authentication, genetic-stability testing, batch-to-batch consistency, validated potency assays, and predefined control procedures for containment and rescue [279, 280]. In parallel, early-phase trials should evaluate not only radiographic response, but also localization biomarkers, persistence and clearance profiles, immune activation signatures, shedding risk, and the interaction of bacterial therapy with antibiotics, chemotherapy, radiation, and checkpoint blockade [244, 279]. These requirements may seem demanding, but they are precisely what will determine whether bacterial therapeutics can move beyond compelling preclinical biology toward reproducible and safe clinical implementation. The long-term success of the bacterial-based approaches will therefore depend as much on translational discipline and standardization as on further innovation in bacterial engineering itself [280, 281].

In summary, bacterial-based cancer therapy represents a distinctive frontier in precision oncology because it combines biological targeting with therapeutic programmability. Its future impact will depend less on the simple claim that bacteria can kill tumors and more on whether the field can define generalizable design principles linking bacterial mechanisms, tumor phenotypes, safety control, and rational therapeutic combinations. When framed in this way, bacterial therapeutics are not merely experimental curiosities, but plausible components of mechanism-based cancer treatment strategies.

## Data Availability

Not applicable.
